# Long‐Term Secondary Preventive Medication Persistence and Adherence in Young Ischemic Stroke Survivors: A Prospective Single‐Center Cohort Study

**DOI:** 10.1002/brb3.71248

**Published:** 2026-01-30

**Authors:** Qiqi Wang, Mingyu Tang, Haiquan Gao, Yuhui Sha, Ming Yao, Yicheng Zhu, Bin Peng, Lixin Zhou, Jun Ni

**Affiliations:** ^1^ Department of Neurology, State Key Laboratory of Complex Severe and Rare Diseases, Peking Union Medical College Hospital Peking Union Medical College and Chinese Academy of Medical Sciences Beijing China

**Keywords:** long‐term outcomes, medication adherence, secondary prevention, young ischemic stroke

## Abstract

**Introduction:**

The study aimed to evaluate long‐term medication persistence and adherence to secondary prevention therapies in young ischemic stroke survivors (aged 18–49 years) and identify factors influencing these outcomes.

**Methods:**

The single‐center prospective cohort study enrolled young ischemic stroke patients (aged 18–49 years) from March 2017 to March 2023. Medication persistence (continuation of all prescribed secondary prevention drugs) and adherence (assessed by the Morisky Medication Adherence Scale‐8 (MMAS‐8)) were evaluated, with reasons for discontinuation and influencing factors analyzed.

**Results:**

Among 226 patients (median age 35 years, 34.5% female), 80.1% remained persistent with their medication regimen over a median follow‐up of 3.9 years. Patients with persistence had higher rates of large artery atherosclerosis (42% vs. 22.2%, *p* = 0.015) and comorbid diabetes (13.3% vs. 2.2%, *p* = 0.015). The median MMAS‐8 score was 7 (6–7.38), with 24.2% showing high adherence, 63% moderate adherence, and 12.8% poor adherence. Poor adherence was associated with younger age (<35 years, *p* = 0.018), the absence of large artery atherosclerosis (*p* = 0.017), and a lower quality of life (*p* = 0.004). Multivariate analysis revealed that older age (*p* = 0.043) and large artery atherosclerosis (*p* = 0.047) were independent predictors of better adherence.

**Conclusion:**

Young ischemic stroke patients demonstrated high medication persistence and moderate adherence, which were influenced by age, stroke etiology, and quality of life. These findings highlight the need for tailored secondary prevention strategies to improve outcomes in this population.

## Graphical abstract

In a prospective cohort of 226 young ischemic stroke survivors, medication persistence was high (80.1%) over a median 3.9‐year follow‐up, while adherence was moderate. Older age and large artery atherosclerosis predicted better adherence, whereas younger age and lower quality of life were associated with poor adherence.

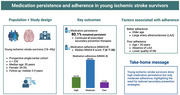

**Abbreviations**
AF: Atrial Fibrillation
AFL: Atrial Flutter
CAD: Coronary Heart Disease
CI: Confidence Interval
GBD: The Global Burden of Disease
IQR: Interquartile Range
MMAS‐8: Morisky Medication Adherence Scale‐8
mRS: The modified Rankin Scale
OR: Odds Ratio
PFO: Patent Foramen Ovale
SS‐QOL: Stroke‐Specific Quality of Life Scale
STROBE: Strengthening the Reporting of Observational Studies in Epidemiology
TIA: Transient Ischemic Attack
TOAST: The Trial of Org 10172 in Acute Stroke Treatment

## 1 Introduction

The Global Burden of Disease (GBD) study revealed that the incidence and burden of stroke, one of the leading causes of death and disability worldwide, increased from 1990 to 2021 (GBD 2021 Stroke Risk Factor Collaborators 2024, 202). Stroke is traditionally considered a disease of elderly individuals, and it is now increasingly affecting younger populations. Young ischemic strokes account for 10% to 14% of all ischemic strokes (Nedeltchev et al., 2005; Putaala et al., 2009; Kappelle et al., 1994; von Sarnowski et al., 2013; Williams et al., 1997; Ferro et al., 2010; Rutten‐Jacobs et al., 2011), and their incidence has risen by 40% since the 1980s (George et al., 2011; Vangen‐Lønne et al., 2015). This trend is even more pronounced in China. Compared with elderly stroke patients, young stroke patients face greater challenges in terms of recurrence risk, long‐term medication adherence, and quality of life.

The consistent use of secondary prevention medications, including antithrombotic agents, statins, and antihypertensive drugs, is crucial for reducing stroke recurrence and improving outcomes in patients with ischemic stroke, with medication adherence being a key factor (Bushnell et al., 2011). Studies have shown that poor medication adherence is significantly associated with increased risks of stroke recurrence, disability, and mortality (Dalli et al., 2021; Zhang et al., 2021). However, existing research has focused primarily on elderly patients, with few studies examining medication adherence in young stroke patients. Previous data suggest that medication adherence is generally low among young stroke patients (Sung et al., 2017). Young patients often face unique physiological, psychological, and social needs and often face multiple pressures from work, family responsibilities, and social activities, which may impact their medication adherence.

Therefore, it is essential to explore the factors that influence medication adherence in young stroke patients to develop more tailored management and intervention strategies. This study aims to analyze medication persistence and adherence and the influencing factors in a prospective cohort of young ischemic stroke patients, with a focus on secondary prevention medications. Additionally, this study sought to explore the correlation between medication adherence and functional outcomes as well as quality of life in young ischemic stroke patients, providing a foundation for future management strategies for this population.

## 2 Materials and Methods

### 2.1 Study Population

**FIGURE 1:**
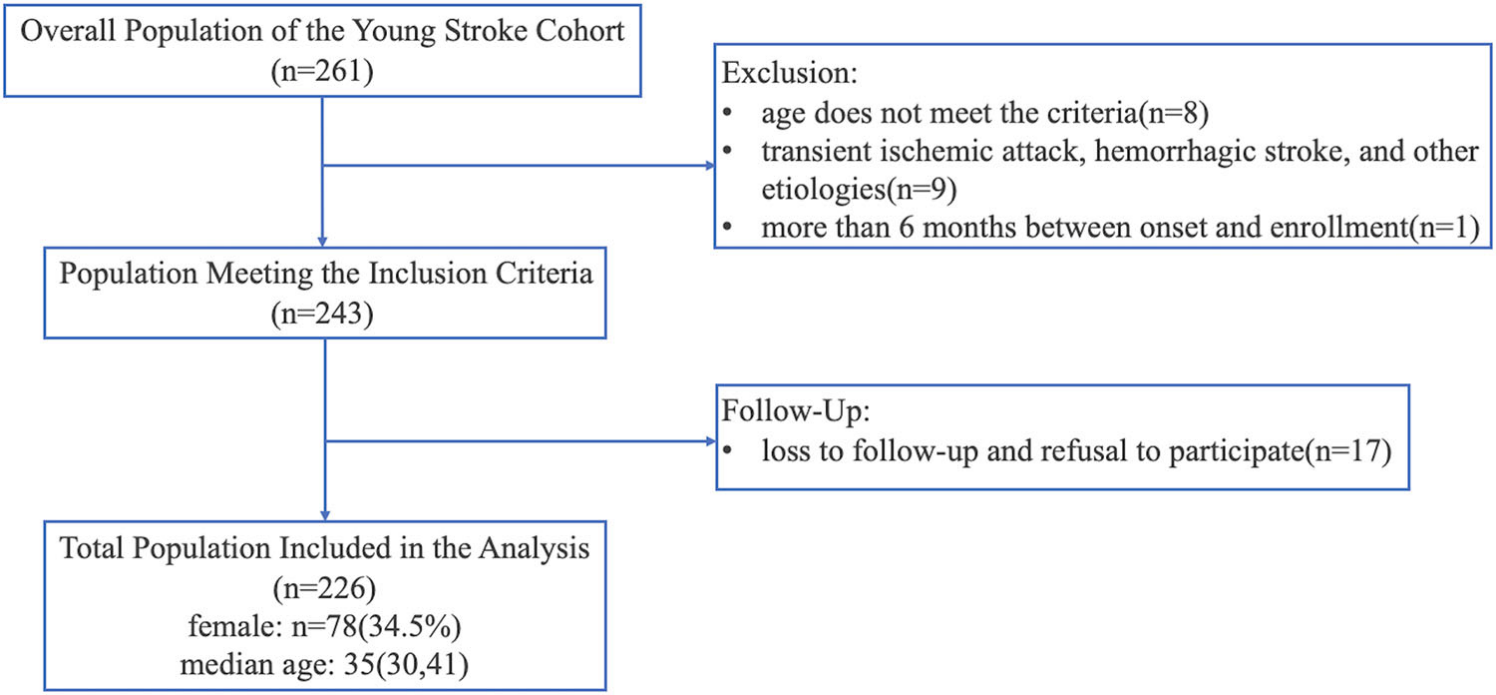
Enrollment flowchart

We prospectively and consecutively enrolled young ischemic stroke patients who visited Peking Union Medical College Hospital from March 2017 to March 2023 and conducted a cross‐sectional follow‐up study. The inclusion criteria were as follows: (1) acute ischemic stroke occurring within the past 6 months; (2) age at onset between 18 and 49 years; (3) a minimum of 1 year between enrollment and follow‐up; and (4) provision of signed informed consent. The exclusion criteria were as follows: (1) transient ischemic attack (TIA), hemorrhagic stroke, or venous infarction; (2) stroke caused by surgical or vascular interventional procedures, such as carotid endarterectomy, angiography, stenting, or cardiac surgery; (3) stroke directly caused by trauma; and 4) inability to complete follow‐up as required during the study period. (Figure 1)

This study followed the Strengthening the Reporting of Observational Studies in Epidemiology (STROBE) guidelines and was approved by the Ethics Committee of Peking Union Medical College (Reference Number: JS‐1281). After a thorough explanation of the study protocol, all participants provided written informed consent.

### 2.2 Study Design

The baseline data for this study were derived from the Etiology and Risk Factors Cohort of Young Stroke Patients and included demographic characteristics, etiology, stroke severity, medical history, risk factors, and medication use. The demographic characteristics included age, sex, marital status, ethnicity, and years of education. The risk factors included a history of ischemic stroke/TIA, hypertension, diabetes, hyperlipidemia, coronary heart disease (CAD), atrial fibrillation (AF)/atrial flutter (AFL), smoking, alcohol consumption, patent foramen ovale (PFO), and migraine. A family history of early‐onset stroke was defined as a history of stroke in first‐ or second‐degree relatives with an onset age between 18 and 49 years. The modified Rankin scale (mRS) (Farrell et al., 1991) was used to assess baseline disability caused by the current stroke at enrollment. The Trial of Org 10172 in Acute Stroke Treatment (TOAST) classification (Adams et al., 1993) was applied to categorize the etiology of ischemic stroke. Secondary preventive medications included antithrombotic agents (antiplatelet drugs and anticoagulants), statins, and antihypertensive drugs.

### 2.3 Follow‐Up Procedures

From August 2023 to March 2024, we conducted in‐person follow‐ups with young stroke patients at the hospital and collected prognostic information through standardized, structured questionnaires. For patients unable to attend in person, telephone follow‐ups were arranged. The follow‐up data included recurrent ischemic stroke or TIA, death, mRS score, quality of life, return to work status, use of secondary prevention medications (including antithrombotic agents, statins, and antihypertensive drugs), and medication adherence.

Quality of life was assessed using both a generic and a stroke‐specific instrument: the EuroQol five‐dimension five‐level questionnaire (EQ‐5D‐5L) (Herdman et al., 2011; Luo et al., 2017) and the Stroke‐Specific Quality of Life Scale (SS‐QOL) (Williams et al., 1999; Zhang et al., 2010). The EQ‐5D‐5L is a widely used generic health‐related quality‐of‐life instrument applicable to both general and disease‐specific populations. It evaluates health status across five dimensions: mobility, self‐care, usual activities, pain/discomfort, and anxiety/depression, each with five levels of severity ranging from no problems to extreme problems. In addition, the EQ visual analogue scale (EQ‐VAS) records patients’ self‐rated overall health on a scale from 0 (worst imaginable health) to 100 (best imaginable health). The EQ‐5D‐5L has been validated in stroke populations and shows good reliability and responsiveness. The SS‐QOL is a stroke‐specific quality‐of‐life instrument developed to capture functional and psychosocial domains particularly relevant to stroke survivors. It consists of 49 items across 12 domains, including energy, family roles, language, mobility, mood, personality, self‐care, social roles, thinking, upper extremity function, vision, and work/productivity. Each item is rated on a 5‐point Likert scale, with higher scores indicating better quality of life. The SS‐QOL has been validated in Chinese stroke populations and demonstrates good psychometric properties.

Medication persistence was determined by comparing baseline medication use with patient‐reported current medication use. Patients who reported discontinuation were required to ascertain the reasons, including discontinuation on the basis of medical advice, side effects, concerns about side effects, belief that medication was unnecessary, lack of understanding about the medication, distrust in doctors, and other reasons. In this study, persistence was defined as the continuation of all secondary preventive medications prescribed at the time of baseline enrollment.

Medication adherence was assessed via the Morisky Medication Adherence Scale‐8 (MMAS‐8)^1^. The Chinese version of the MMAS‐8 (Supplementary Table S1) has been widely used in China for evaluating medication adherence in patients with various chronic diseases and has demonstrated good reliability and validity (Morisky et al., 2008; Berlowitz et al., 2017; Bress et al., 2017; Yan et al., 2014; Shi et al., 2022; Yang et al., 2014; Wu et al., 2018). The total score ranges from 0 to 8, with 8 indicating high adherence, 6 ∼ <8 indicating moderate adherence, and <6 indicating low adherence. Events, assessment scales, and medication use were classified by trained assessors and reviewed by experts in the relevant fields.

### 2.4 Data Analysis and Statistics

Given the skewed distribution of the data, continuous variables are described as medians and interquartile ranges (IQRs). Categorical variables are presented as frequencies (*N*). The Pearson chi‐square test was used for categorical variables, and the Mann‒Whitney *U* test was employed to compare continuous variables between groups. Multivariate logistic regression analysis was conducted to evaluate the independent influencing factors for return‐to‐work status and medication adherence among young ischemic stroke patients. Patients were divided into groups on the basis of their return‐to‐work status and medication use. Univariate analysis via the Pearson chi‐square test or Mann‒Whitney *U* test was performed to identify factors with significant differences between groups. Variables showing statistical significance in the univariate analysis were then included as covariates in the multivariate logistic regression model (using a stepwise selection procedure). Statistical analysis was performed via SPSS 26.0 software, with the significance level set at *p* < 0.05 and a confidence interval (CI) of 95%.

Age was categorized using 35 years as the cutoff point. This threshold was chosen because it corresponded to the median age of the study cohort, ensuring balanced group sizes for comparison. In addition, prior studies have suggested that younger and relatively older young stroke patients may differ in stroke etiology, social roles, and health‐related behaviors (Putaala et al., 2009; Verburgt et al., 2024). Categorization of age also facilitated clinical interpretability of the results.

## 3 Results

### 3.1 Baseline Information

The study cohort included 243 young ischemic stroke patients, with 17 patients (7.0%) lost to follow‐up or who refused to participate. Ultimately, 226 (93.0%) young ischemic stroke patients were included in the analysis. The median age was 35 years (IQR, 30, 41), with 78 females (34.5%). The majority of patients (95.1%) were of Han ethnicity, and 172 (76.1%) were married. The average duration of education was 14 years (IQR, 10, 16). The median mRS score at enrollment was 1 (IQR, 0, 1). The median follow‐up duration was 3.9 years (IQR, 2.6, 4.8).

The most common risk factors were a history of smoking in 95 patients (42%), hypertension in 68 patients (30.1%), and hyperlipidemia in 61 patients (29.3%). A total of 137 young ischemic stroke patients (60.6%) had two or more risk factors. According to the TOAST classification, the most common etiology was large artery atherosclerosis, accounting for 86 cases (38.1%), followed by other determined etiologies in 65 cases (28.8%), undetermined etiologies in 38 cases (16.8%), small vessel occlusions in 20 cases (8.8%), and cardioembolisms in 17 cases (7.5%). With respect to baseline secondary prevention treatment for ischemic stroke, 88.5% (200 patients) received antiplatelet therapy, 7.5% (17 patients) received anticoagulation therapy, 90.3% (204 patients) received statin therapy, and 23.9% (54 patients) received antihypertensive therapy. The detailed baseline information of the patients is presented in Table 1.

**TABLE 1:** Baseline characteristics of young ischemic stroke patients.

	All patients (*n* = 226) *N*%/IQR		All patients (*n* = 226) *N*%/IQR
Female	78 (34.5%)	Age	35 (30, 41)
Ethnicity: Han	215 (95.1%)	Married	172 (76.1%)
Years of education	14 (10,16)	Occupation: Professional and technical personnel	74 (32.7%)
Enrollment mRS^a^ Score	1 (0,1)		
**Risk factors**			
Smoking history	95 (42.0%)	Hypertension	68 (30.1%)
Diabetes	23 (10.2%)	CAD^b^	3 (1.3%)
Hyperlipidemia	61 (29.3%)	Family history of early‐onset stroke	33 (15.9%)
AF/AFL^c^	3 (1.3%)	PFO^d^	6 (2.7%)
Migraine	10 (4.4%)	Alcohol consumption history	75 (33.2%)
History of TIA^e^	20 (8.8%)	History of cerebral infarction	58 (2.6%)
Presence of 2 or more risk factors	137 (60.6%)	Number of risk factors	2 (1, 3)
**TOAST** ^f^ **classification**			
Large‐artery atherosclerosis	86 (38.1%)	Cardioembolism	17 (7.5%)
Small artery occlusion	20 (8.8%)	Other determined etiology	65 (28.8%)
Undetermined etiology	38 (16.8%)		
**Secondary prevention pharmacotherapy**			
Antiplatelet agents	200 (88.5%)	Anticoagulants	17 (7.5%)
Statins	204 (90.3%)	Antihypertensive drugs	54 (23.9%)

^a^ *mRS*: modified Rankin scale

^b^ *CAD*: coronary heart disease

^c^ *AF*: atrial fibrillation, *AFL*: atrial flutter

^d^ *PFO*: patent foramen ovale

^e^ *TIA*: transient ischemic attack

^f^ *TOAST*: Trial of Org 10172 in Acute Stroke Treatment Classification

### 3.2 Analysis of Medication Persistence and Influencing Factors

During the follow‐up period, 181 (80.1%) young ischemic stroke patients persisted with their medication, whereas 45 patients (19.9%) discontinued at least one secondary prevention medication. The highest persistence rate was observed for anticoagulants (100%), followed by antiplatelet agents (83.5%), statins (81.9%), and antihypertensive drugs (79.6%).

Among the 45 (19.9%) young stroke patients who discontinued at least one secondary preventive medication, the reasons for discontinuation were investigated. Twenty patients (44.4%) believed that they no longer needed medication, followed by 10 patients (22.2%) because of adverse drug reactions, 9 patients (20%) because of medical advice, and 2 patients (4.4%) because of concerns about potential adverse reactions.

Patients were divided into groups on the basis of medication persistence. Compared with patients who discontinued medication, patients who persisted had a greater proportion of large artery atherosclerosis (42% vs. 22.2%, *p* = 0.015) and a greater proportion of comorbid diabetes (13.3% vs. 2.2%, *p* = 0.015). However, logistic multivariate regression analysis, incorporating variables such as sex, age, diabetes, and large artery atherosclerosis, did not identify any independent factors influencing medication persistence. (Table 2)

**TABLE 2:** Factors influencing persistence with secondary prevention medications for young stroke patients.

	Univariate analysis			Multivariate analysis	
	Persistence of medication *N* = 181 *N*%/IQR	Discontinuation of medication *N* = 45 *N*%/IQR	*p* ^a^ value	OR^b^ (95%CI)	*p* value
Female	57 (31.5%)	21 (46.7%)	0.055	0.314 (0.088, 1.118)	0.074
Age (≥35 years)	99 (54.7%)	20 (44.4%)	0.218	0.364 (0.085, 1.553)	0.172
Ethnicity: Han	172 (96.6%)	43 (95.6%)	0.79		
Married	141 (79.2%)	31 (68.9%)	0.175		
Educational level: primary school or below	18 (11%)	3 (7.3%)	0.483		
Years of education	15 (11, 16)	14 (9.5, 16)	0.717		
Occupation: Professional and technical personnel	57 (32.8%)	17 (38.6%)	0.103		
**TOAST Classification**					
Large‐artery atherosclerosis	76 (42%)	10 (22.2%)	**0.015**	0.438 (0.115, 1.676)	0.228
Cardioembolism	11 (6.1%)	6 (13.3%)	0.115		
Small artery occlusion	18 (9.9%)	2 (4.4%)	0.38		
Other determined etiology	48 (26.5%)	17 (37.8%)	0.135		
Undetermined etiology	28 (15.5%)	10 (22.2%)	0.278		
**Risk factors**					
Smoking history	76 (42%)	19 (42.2%)	0.977		
Hypertension	57 (31.5%)	13 (28.9%)	0.735		
Diabetes	24 (13.3%)	1 (2.2%)	**0.015**	2.6 (0.283, 23.9)	0.398
Hyperlipidemia	55 (30.4%)	8 (17.8%)	0.091		
CAD	5 (2.8%)	0	0.586		
Family history of early‐onset stroke	29 (16%)	6 (13.3%)	0.655		
Alcohol consumption history	59 (32.6%)	16 (35.6%)	0.706		
History of TIA	19 (10.5%)	2 (4.4%)	0.176		
History of cerebral infarction	48 (26.5%)	11 (24.4%)	0.777		
AF/AFL	3 (1.7%)	0	1		
PFO	5 (2.8%)	1 (2.2%)	0.837		
Migraine	7 (3.9%)	3 (6.7%)	0.437		
≥2 risk factors	108 (59.7%)	29 (64.4%)	0.557		
**Prognosis**					
Admission mRS score (≤1)	19 (11.9%)	3 (6.8%)	0.421		
Follow‐up mRS score (≤1)	13 (7.2%)	1 (2.2%)	0.312		
Recurrence of stroke events	22 (12.2%)	3 (6.7%)	0.268		
EQ‐5D‐5 L	1 (0.91, 1)	0.98 (0.94, 1)	0.114		
SS‐QOL^c^	243 (238.5,245)	244 (235.5, 245)	0.584		
Return to work	163 (90.6%)	42 (93.3%)	0.771		

^a^ *P* value of the Mann–Whitney *U* test for continuous variables and chi‐square test for categorical variables. A *p*‐value of less than 0.05 was considered statistically significant

^b^ The odds ratios (ORs) were adjusted for sex, age, large artery atherosclerotic stroke, and diabetes.

^c^ *SS‐QOL*: Stroke‐Specific Quality of Life Scale

Fisher's exact test was applied for a sample size ≤5 in either category.

### 3.3 Medication Adherence and Influencing Factors

In terms of medication adherence, the median MMAS‐8 score was 7 (6, 7.38). Among the patients, 24.2% (53 patients) demonstrated high medication adherence (MMAS‐8 score of 8), 63% (138 patients) showed moderate adherence (MMAS‐8 score of 6–7), and 12.8% (28 patients) showed poor adherence (MMAS‐8 score <6).

Patients were divided into two groups on the basis of whether their MMAS‐8 score was <6. Compared with the moderate‐ and high‐adherence groups, the low‐adherence group had a lower proportion of patients aged 35 years or older (32.1% vs. 56%, *p* = 0.018), a lower proportion of patients with large artery atherosclerosis (17.9% vs. 41.4%, *p* = 0.017), and lower overall SS‐QOL scores (235 vs. 244, *p* = 0.004) (Table 3).

**TABLE 3:** Factors influencing medication adherence in young stroke patients

	Univariate analysis			Multivariate analysis	
	Moderate to high adherence *N* = 191 *N*%/IQR	Low adherence *N* = 28 *N*%/IQR	*p* ^a^ value	OR^b^ (95%CI)	*p‐*value
Female	64 (33.5%)	10 (35.7)	0.818	0.776 (0.317, 1.901)	0.58
Age (≥35 years)	107 (56%)	9 (32.1%)	**0.018**	**0.407 (0.17, 0.971)**	**0.043**
Ethnicity: Han	181 (95.8%)	27 (100%)	0.552		
Married	148 (78.3%)	19 (70.4%)	0.205		
Educational level: primary school or below	20 (11.5%)	1 (4.3%)	0.297		
Years of education	14 (10, 16)	15 (11, 16)	0.531		
Occupation: Professional and technical personnel	65 (35.1%)	9 (34.6%)	0.254		
**TOAST classification**					
Large‐artery atherosclerosis	79 (41.4%)	5 (17.9%)	**0.017**	**0.344 (0.12, 0.987)**	**0.047**
Cardioembolism	13 (6.8%)	3 (10.7%)	0.481		
Small artery occlusion	17 (8.9%)	3 (10.7%)	0.761		
Other determined etiology	55 (28.8%)	9 (32.1%)	0.716		
Undetermined etiology	27 (14.1%)	8 (28.6%)	0.069		
**Risk factors**					
Smoking history	82 (42.9%)	12 (42.9%)	0.994		
Hypertension	60 (31.4%)	8 (28.6%)	0.761		
Diabetes	22 (11.5%)	1 (3.6%)	0.15		
Hyperlipidemia	55 (28.8%)	6 (21.4%)	0.417		
CAD	3 (1.6%)	0	1		
Family history of early‐onset stroke	28 (14.7%)	4 (14.3%)	0.958		
Alcohol consumption history	61 (31.9%)	11 (39.3%)	0.44		
History of TIA	18 (9.4%)	2 (7.1%)	0.687		
History of cerebral infarction	48 (25.1%)	8 (28.6%)	0.697		
AF/AFL	2 (1%)	1 (3.6%)	0.338		
PFO	6 (3.1%)	0	1		
Migraine	7 (3.7%)	2 (7.1%)	0.424		
≥2 risk factors	117 (61.3%)	16 (57.1%)	0.677		
**Prognosis**					
Admission mRS score (≤1)	150 (88.8%)	26 (96.3%)	0.18		
Follow‐up mRS score (≤1)	180 (94.2%)	26 (92.9%)	0.778		
Recurrence of stroke events	20 (10.5%)	3 (10.7%)	0.969		
EQ‐5D‐5 L	1 (0.942, 1)	0.951 (0.893, 1)	0.171		
SS‐QOL	244 (239, 245)	235 (222, 245)	**0.004**	0.989 (0.975, 1.003)	0.129
Return to work	173 (90.6%)	27 (96.4%)	0.255		
**Secondary prevention pharmacotherapy**					
Antiplatelet agents	174 (91.1%)	25 (89.3%)	0.761		
Anticoagulants	15 (7.9%)	2 (7.1%)	0.894		
Statins	176 (92.1%)	27 (96.4%)	0.376		
Antihypertensive drugs	50 (26.2%)	4 (14.3%)	0.173		

^a^ *p*‐value of the Mann–Whitney *U* test for continuous variables and chi‐square test for categorical variables. A *p‐*value of less than 0.05 was considered statistically significant.

^b^ Odds ratio (OR) values adjusted for sex, age, large artery atherosclerotic stroke, and SS‐QOL.

Fisher's exact test was applied for a sample size ≤5 in either category.

Multivariate logistic regression analysis, which included variables such as sex, age, large artery atherosclerosis, and SS‐QOL, revealed that age ≥35 years (*p* = 0.043, OR = 0.407, 95% CI: 0.17–0.971) and large artery atherosclerosis (*p* = 0.047, OR = 0.344, 95% CI: 0.12–0.987) were independently associated with moderate to high medication adherence in young stroke patients.

## 4 Discussion

This study is a bidirectional cohort study evaluating the persistence of and adherence to secondary prevention medications in young ischemic stroke patients. Through an average follow‐up of 4 years, we found that 80.1% of patients continued to use secondary prevention medications. The overall medication adherence among young ischemic stroke patients was moderate, with 12.8% exhibiting poor adherence. Poor medication adherence was positively correlated with age <35 years and the absence of large artery atherosclerosis, and it was significantly associated with lower quality of life. This study highlights the importance of addressing medication adherence and persistence, along with the factors that influence them, in secondary prevention strategies for young adults with ischemic stroke.

Over the past two decades, significant advancements have been made in the secondary prevention of ischemic stroke, including antithrombotic therapy, statin therapy, and risk factor control. Standardized medication regimens and good adherence are crucial for preventing recurrence and reducing disability and mortality (Dalli et al., 2021; Zhang et al., 2021). However, the impact of these advancements on young ischemic stroke patients has not been fully explored. Owing to the complex etiology and diagnostic challenges of young patients with ischemic stroke, there is a lack of targeted prevention guidelines (Kim et al., 2023). The use of medication and adherence among young ischemic stroke patients remain challenging in clinical practice. A study in Taiwan revealed that among many young stroke patients with preexisting conditions such as hypertension, diabetes, or hyperlipidemia, 71.5%, 64.3%, and 88.4%, respectively, did not adhere to medications after stroke (Sung et al., 2017). Similarly, one study reported that less than half of young ischemic stroke patients take statins, although persistent statin use is linked to reduced mortality and stroke recurrence (van Dongen et al., 2019). Several recent studies have shown that the discontinuation of antiplatelet and antihypertensive medications, as well as the irregular use of antihypertensive drugs, is associated with an increased risk of recurrent stroke and mortality in young adults (Pezzini et al., 2014; van Dongen et al., 2019). With the increasing incidence of stroke in young individuals and the growing focus on young ischemic stroke patients, clinicians are increasingly emphasizing secondary prevention medications and patient education for this population. Our study revealed that 80.1% of young ischemic stroke patients continued taking medication during an average of 4 years of follow‐up. Adherence to secondary prevention medications was moderate, which was consistent with previous findings. The study suggests that regular follow‐up and medication guidance by stroke specialists contribute to higher rates of medication persistence and adherence. Long‐term follow‐up and personalized secondary prevention strategies may constitute the optimal model for improving medication adherence in young ischemic stroke patients.

This study did not find a correlation between persistent medication use or high medication adherence and stroke recurrence. Previous studies have shown that the etiology of young ischemic stroke is more complex than that of stroke in older adults and that the risk of recurrence varies significantly depending on the underlying cause. For example, large artery atherosclerosis and cardioembolic stroke are associated with a greater risk of recurrence, whereas cryptogenic stroke is associated with a lower risk of recurrence (Verburgt et al., 2024). In this study, large artery atherosclerosis was the most common etiology, and patients with this type of stroke were more likely to persist with medication and exhibit higher adherence, which may have contributed to a reduced overall risk of stroke recurrence. This finding raises an important question: should all young ischemic stroke patients receive long‐term secondary prevention medication? Recent studies have reported that the risk of hemorrhagic events in young patients over the long term is nearly comparable to the risk of ischemic events (Verhoeven et al., 2022). Therefore, for young patients with a low risk of recurrent ischemic events, the benefits of antithrombotic therapy may not always outweigh the risks of bleeding. The benefits of persistent antithrombotic therapy and the safety of discontinuing such therapy in young stroke patients with specific etiologies remain unclear. Given the uncertainty surrounding stroke recurrence risk and secondary prevention decisions in young patients, further research, particularly randomized controlled trials, is needed to clarify the long‐term benefits and risks of secondary prevention medications in young stroke patients with different etiologies.

Previous studies have shown that medication adherence in elderly stroke patients is influenced by various factors, such as sex, age, education level, economic status, stroke severity, and stroke etiology (Jiang et al., 2017; Chen et al., 2024; Hoarau et al., 2024). However, young adults face unique challenges that may impact medication persistence and adherence, including the need for long‐term medication use, pressure to return to work and social life, and differences in disease understanding. Our study revealed that the factors influencing medication adherence in young stroke patients differ significantly from those in elderly stroke patients. Traditional socioeconomic factors, such as education level and occupation, do not affect medication adherence in young stroke patients. The etiology of large artery atherosclerosis was associated with increased medication persistence and adherence, likely due to severe vascular stenosis and defined secondary prevention plans. Additionally, we found that patients younger than 35 years old had poorer medication adherence. This may be attributed to several factors: first, the etiology of stroke in young patients is often unclear or lacks targeted treatment options; second, younger patients tend to have better functional outcomes after stroke, leading them to believe that further preventive medication is unnecessary; third, many young patients return to work after stroke, and their busy lifestyles may result in missed doses; and fourth, a significant proportion of patients under 35 years of age have fertility concerns. These findings highlight the need for personalized medication plans and educational approaches, such as consultations, online seminars, and support groups, to improve adherence and self‐management among young stroke patients.

In this study, the reasons for medication discontinuation revealed that 80% of cases were due to noncompliance with the prescribed regimen. The most common reasons cited by patients were the belief that they no longer needed the medication and concerns about or experiences with adverse drug reactions. This finding highlights the cognitive differences among young stroke patients regarding the importance of medication therapy and their concerns about potential side effects, which is consistent with many previous studies. Consequently, improving patients' understanding of the importance of sustained secondary prevention remains a challenge in the management of ischemic stroke in young adults.

This study revealed that poor medication adherence among young stroke patients was significantly associated with lower quality of life as assessed by the stroke‐specific SS‐QOL, whereas no significant association was observed with the generic EQ‐5D‐5L. This discrepancy may be explained by differences in the conceptual focus and sensitivity of the two instruments. The EQ‐5D‐5L is designed to capture overall health status and may be less sensitive to subtle stroke‐related functional, cognitive, and psychosocial impairments, particularly in younger patients with relatively good global health. In contrast, the SS‐QOL includes stroke‐specific domains that more directly reflect poststroke functional limitations and emotional well‐being, which are likely to influence patients’ perceptions of disease burden and motivation for long‐term medication adherence. These findings suggest that poor medication adherence not only increases the risk of stroke recurrence but also may negatively impact patients' overall health and well‐being. Patients with poor medication adherence often face greater challenges in poststroke rehabilitation, including more severe functional disabilities and psychological distress. To address this issue, future research should focus on exploring strategies to improve medication adherence through patient education and psychological interventions, ultimately enhancing the quality of life of young stroke patients.

### 4.1 Limitations

This study has several limitations: (1) This was a single‐center cohort from a large tertiary hospital. The stroke cohort was originally established to explore etiology and risk factors, and due to loss to follow‐up and refusal to participate, there is a potential for selection bias. (2) The sample size was relatively small, and the median follow‐up duration of 3.9 years was relatively short. Additionally, the varying lengths of follow‐up limit the generalizability of the results. Future studies should include more patients and extend the follow‐up period to enable more comprehensive statistical analysis. (3) The association between quality of life and medication adherence was assessed cross‐sectionally at follow‐up, which precludes causal inference. Lower quality of life may lead to poorer medication adherence, but nonadherence itself may also negatively affect quality of life. Longitudinal studies are needed to clarify the temporal and causal relationships between these factors.

## 5 Conclusion

This study provides insights into medication persistence and adherence to secondary prevention drugs among young ischemic stroke patients, highlighting their relationships with etiology and risk factors. Young ischemic stroke patients presented high rates of long‐term medication persistence and moderate levels of medication adherence. While most patients continued medication regimens during the follow‐up period, multiple reasons, such as adverse drug reactions and misconceptions about medication therapy, led to discontinuation in a proportion of patients. Medication adherence in young stroke patients is influenced by age and stroke etiology and is closely linked to poststroke quality of life. Improving medication adherence in young stroke patients is a complex and multifaceted process involving individual health management, family and social support, simplification of treatment regimens, and psychological and behavioral interventions. In the future, personalized treatment plans, patient education, and advanced technologies such as remote monitoring or artificial intelligence could be employed to increase medication adherence, reduce stroke recurrence, and improve long‐term outcomes in young stroke patients.

## Author Contributions

JN, LZ, MY, YZ, BP: Study concept and design. QW, MT, YS, HG: Data collection and analysis. QW, MT: Drafting the manuscript. JN, LZ: Critical revision for intellectual content. All authors read and approved the final manuscript.

## Funding

This work was supported by the National High Level Hospital Clinical Research Funding (Grant ID: 2022‐PUMCH‐D‐007).

## Ethics Statement

This study was conducted in accordance with the Declaration of Helsinki (https://www.wma.net/policies‐post/wma‐declaration‐of‐helsinki/) and approved by the Institutional Review Board of Peking Union Medical College Hospital (Approval No. JS‐1281). The ethics committee granted a waiver of additional approval for retrospective data analysis where applicable, with all patient data being anonymized prior to analysis.

## Consent

Written informed consent was obtained from all participants after full explanation of the study objectives and procedures.

## Conflicts of Interest

The authors declare no conflicts of interest.

## Supporting information




**Supplementary Material**: brb371248‐sup‐0001‐TableS1.docx

## Data Availability

This research utilizes proprietary data provided by PUMCH. These resources are owned by PUMCH and are not publicly available.

## Endnotes

1 The MMAS‐8 Scale, content, name, and trademarks are protected by US copyright and trademark laws. Permission for use of the scale and its coding is required. A license agreement is available from MMAR, LLC., www.moriskyscale.com.
