# Supplementary material for: Long‐Term Secondary Preventive Medication Persistence and Adherence in Young Ischemic Stroke Survivors: A Prospective Single‐Center Cohort Study
Source: Brain Behav. 2026 Jan 30;16(2):e71248. doi: 10.1002/brb3.71248 (PMC12856368; doi:10.1002/brb3.71248)
Supplement: Supplementary file 1 — Supplementary Material: brb371248‐sup‐0001‐TableS1.docx [file BRB3-16-e71248-s001.docx]

The MMAS-8 Scale

| 1. 1. Do you sometimes forget to take your medicine? | ⬜ _1_NO ⬜ _0_ YES |
| --- | --- |
| 2. People sometimes miss taking their medications for reasons other than forgetting. Thinking over the past 4 weeks, were there any times when you did not take your medication? | ⬜ _1_ NO ⬜ _0_ YES |
| 3. Would you ever cut back or stop taking your medication without telling your doctor because you felt worse when you took it? | ⬜ _1_ NO ⬜ _0_ YES |
| 4. When you travel or leave home, do you sometimes forget to bring along your medication? | ⬜ _1_ NO ⬜ _0_ YES |
| 5. Did you take your medication yesterday or the last time you were supposed to take it? | ⬜ _0_ NO ⬜ _1_YES |
| 6. When you feel that your medical condition is under control, do you sometimes stop taking your medication? | ⬜ _1_ NO ⬜ _0_ YES |
| Taking medication exactly as prescribed is a real inconvenience for some people. Do you ever feel hassled about sticking to your treatment plan? | ⬜ _1_ NO ⬜ _0_ YES |
| 8. How often do you have difficulty remembering to take all of your medications? | ⬜ _0_ ALL OF THE TIME  ⬜ _0.25_ USUALLY  ⬜ _0.5_ SOMETIMES  ⬜ _0.75_ ONCE IN A WHILE  ⬜ _1_ NEVER/RARELY |
